# Inclusion of special populations in clinical research: important considerations and guidelines

**Published:** 2018-04-07

**Authors:** Stuart S. Winter, Janet M. Page-Reeves, Kimberly A. Page, Emily Haozous, Angelica Solares, Carla Nicole Cordova, Richard S. Larson

**Affiliations:** ^1^Children's Minnesota Research Institute, Minneapolis, MN, United States; ^2^Department of Family and Community Medicine, University of New Mexico, Albuquerque, United States; ^3^Department of Internal Medicine, Division of Epidemiology, Biostatistics and Preventive Medicine, University of New Mexico, Albuquerque, United States; ^4^UNM College of Nursing, University of New Mexico, Albuquerque, United States; ^5^University of New Mexico School of Law, University of New Mexico, Albuquerque, United States; ^6^UNM Clinical and Translational Science Center, University of New Mexico, Albuquerque, United States

**Keywords:** clinical trials, special, populations, accrual, retention, best practices

## Abstract

**Background:**

Trials that involve human participants call for experiments or observations that are performed in a clinical research setting. Currently, there are over 16,000 clinical trials open in the United States. Despite continuing efforts to include "special populations" in clinical trials, there are gaps in participation for people who are either minors or elderly adults, are from historically under-represented minorities, or live in rural communities. The inclusion of these special populations in clinical trials research is essential for conclusions that benefit all populations. Data suggest that study partic-ipation rates for special populations have fallen to levels that could endanger the successful performance of some types of research. This is particularly concerning in the 21st century, where demographic trends in the United States continue to shift towards an older and Hispanic population with fewer rural dwellers. Trends in New Mexico and other minority-majority states mirror many of these shifts.

**Relevance for patients:**

In this review, we highlight improvement strategies for enhanced clinical trial participation by members of special populations. Key drivers for disparate clinical trials participation and outcomes often include differences in genetics, physiology, and perceptions of mistrust towards researchers. To overcome these barriers, we focus on best practices in recruitment strategies from the perspectives of the participants, the researchers and the institutions that support clinical trials.

## 1. Introduction

Demographic transitions signify important milestones in the social and scientific evolution of the United States (U.S.). Over the course of the next few decades the U.S. will witness a transition from a predominantly ethnically and racially homogenous society to a more heterogeneous one. By the year 2044 non-Hispanic Whites will no longer enjoy an ethnic majority status, and by the year 2060 Hispanics/Latinos, who are the third fastest growing ethnicity nationally, will account for more than one¬quarter of the total U.S. population. Census reporting of two or more races per individual are expected to increase, with steep declines in non-Hispanic White alone reporting [1]. Concurrent to these ethnic and racial changes, age demographics will also be rapidly evolving. By 2030 the last of the baby boomer generation will turn 65, while overall fertility rates will continue to decline. Despite continuing efforts to include representation of different populations in clinical trials, current participation rates do not accurately represent the diverse constituencies of the U.S. For these reasons, recruitment of special populations is needed to assess and continue to advance health related research. Increased participation helps to ensure that sufficient sample size for ethnicity-specific analyses can be conducted and applicable to the diverse populations that researchers seek to serve [2].

In health research the term "special populations" (Table 1) has been used interchangeably with "vulnerable populations" or "diverse populations". The complicated or inconsistent use of terminology in studies can adversely impact the accuracy or design implementation in clinical trials where under-represented groups are being targeted [2]. The National Institute of Health (NIH) has specifically defined vulnerable populations, with protections afforded to those populations based on the characteristics of each group. In human research the vulnerable populations comprised of unborn children (Subpart B), prisoners (Subpart C), children (Subpart D), and those with cognitive impairment have been afforded additional protections, because they are at risk for undue influences in a research environment. The term "diverse populations" has been used to describe women, historically under-represented minorities, and members of the LGBTQ+ community or other populations that sometimes are overlooked in clinical research studies.

**Table 1.**
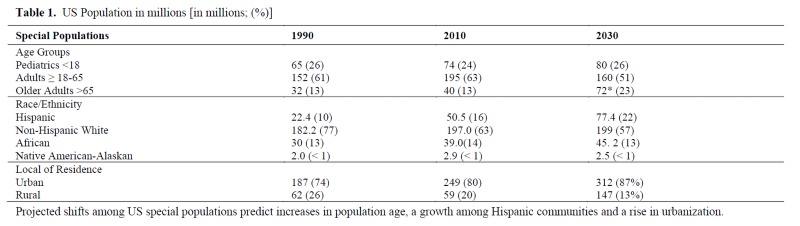
US Population in millions [in millions; (%)] Projected shifts among US special populations predict increases in population age, a growth among Hispanic communities and a rise in urbanization.

**Figure 1.**
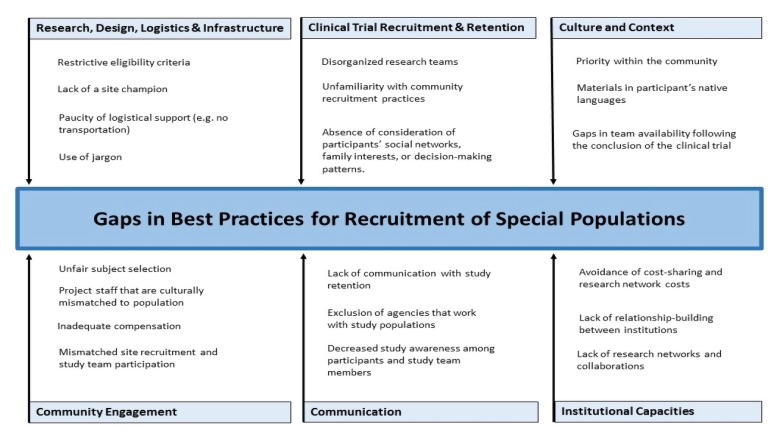
Causative features for gaps in best practices for clinical trial inclusion. Clinical trials recruitment and retention are challenged by at least six areas of deficiencies. A partial listing of specific items is addressed but may be overcome by focused interventions.

For this summary paper, we define "special populations" by age (minors younger than eighteen years of age or elderly adults older than sixty-five), historically under-represented ethnic or racial groups, and people who live in rural areas. Including "special populations" in health research has become recognized as a priority by health care providers, researchers, funders, and community members. However, including special populations can present significant challenges for recruitment and retention of participants. In recent years, there has been attention to this issue in the health research literature. New Mexico mirrors the changing trends in demographics, especially in growth of Native American and Latino populations, many of whom reside in rural, under-served locations. As clinical and translational health research is expanding, our experiences, largely drawn from the UNM Clinical and Translational Science Center (CTSC), can inform this 'comprehensive' understanding and best practices. It is often necessary to engage in multiple, simultaneous strategies including both those intended to generally improve inclusivity and those designed specifically for research with special populations. Such strategies include a complex interplay between research design, logistics and infrastructure, participant recruitment and retention, culture and context, institutional capacity, communication among team members, and community engagement (Figure 1) [3].

## 2. Factors in special populations research and why they matter

Key factors for special population research include differences in genetics and physiology between ethnically and racially defined groups, access to clinical trials for citizens living in rural cultural areas, age-defined variations across the human lifespan and cultural diversity. Historical and contextual matters are discussed as well. Subject sampling is one of the foundational principles in the conduct of well-designed clinical trials. When special populations have been included into clinical trials, numerous age-dependent, community, cultural and genetic features have come to light (Table 2). These key drivers of variance between special populations require consideration when designing clinical trials to answer specific, population-based questions based upon age, racial/ethnic diversity and context.

## 2.1 Age-specific variances in clinical trials outcomes

At either end of the human lifespan, drugs are differentially metabolized, depending upon enzymatic efficiency and organ maturity, as demonstrated by differences in renal, hepatic, and other organ toxicities between infants and adults [4,5]. Moreover, drug studies in children require different research metrics and endpoints that are unrelated to consent/assent in special populations that are defined by age [6,7]. Infants are at particularly increased risk from differences in physiology and organ maturity. Older adults are at increased risk for age-associated adverse events including those related to cardiovascular health, immune function, neuropathies and comorbidity in general. In some cases, barriers to recruitment are created by researchers themselves, as demonstrated by studies that failed to accrue target populations of elderly adults due to over-use of co-morbid exclusion criteria. When comorbidities are used as exclusion criteria, many geriatric patients may not be eligible for studies that they would have otherwise been engaged in as potential participants.

## 2.2 Genetic variances among racial/ethnic groups

Race and ethnicity have been linked to differences in genetic predispositions to disease [8-11]. Now that targeted therapies and personalized approaches to diseases having gene-based variances have become more available, genomically-informed approaches are increasingly important among special populations [12-15]. Members of special populations that are defined by race and ethnicity harbor genetic differences that have biological consequences. Examples of such findings have been described for members of various racial/ethnic groups who received treatment for HIV with efavirenz resulting in better clinical care for these groups [16].

## 2.3 History, context and the ephemeral nature of trust

Knowing the historical and experiential context for a special population can help researchers be aware of issues related to trust that influence participant attitudes and behavior. Trust may be breached by damaging stereotypes are perpetuated by researchers or the research process, and internalized negative messages that influence choices and behaviors among members of that population [17,18]. Killien et al. [19] discuss how unethical research practices influence distrust among women of color. The Tuskegee syphilis study is widely recognized for the residual mistrust that was engendered, not only among African American men and the African American community in general, but also among women partners of the men involuntarily studied who were exposed to syphilis without their knowledge and also not provided with treatment. People of color have been routinely targeted by ideologically driven and unethical trials that involved failure to disclose sterilization, drug testing, or use of biological materials for other purposes [20,21]. This history encourages conspiracy theories about the AIDS epidemic, concepts of genocide, and distrust of researchers; contexts cannot be understated. Choi et al. [22] emphasize the need to build trust and respect and to facilitate a non-threatening environment for participants in a research study.

## 2.4 Access, awareness and geographic isolation

2.4

Access to health care and health awareness may vary between urban and rural populations, affecting behavioral outcomes within communities [23-25]. In many cases, when made aware of differences between special populations, investigators have assessed risks differently and, with better-informed hypotheses, have discovered novel, unexpected mechanisms among the conditions studied (Figure 1).

**Table 2.**
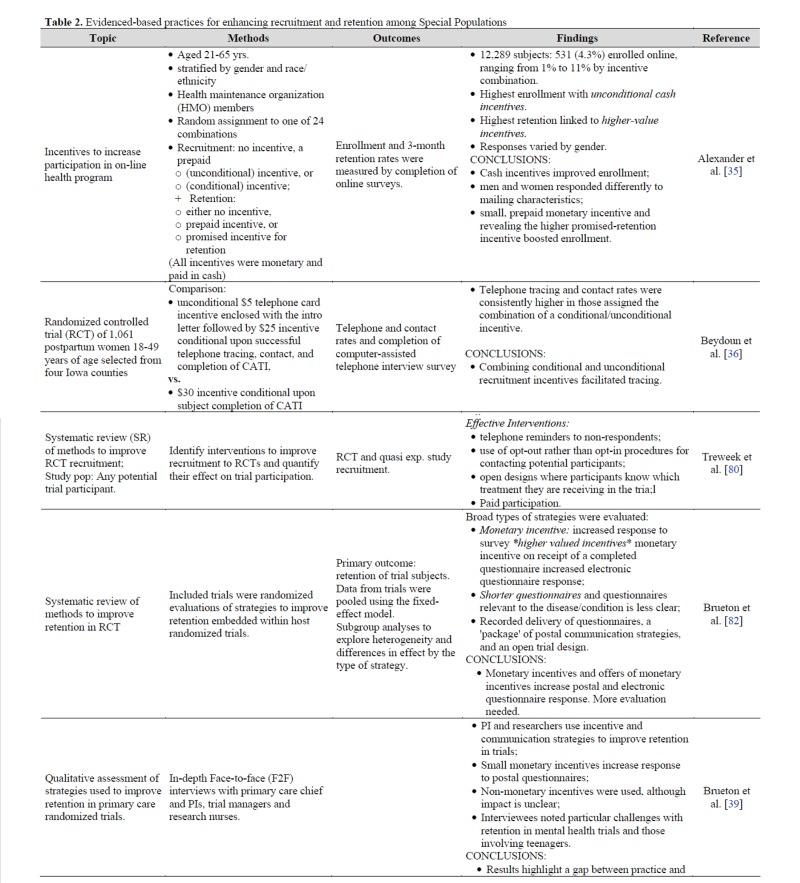

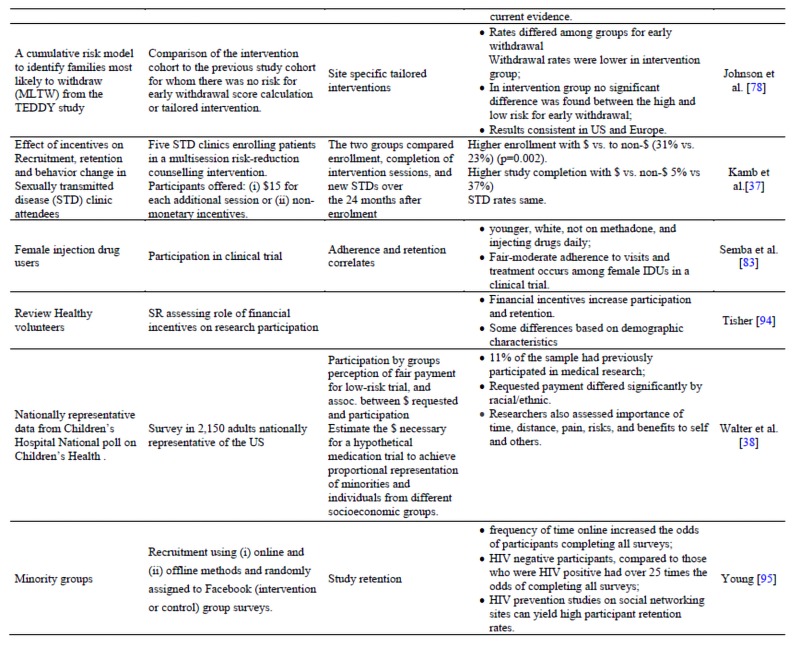
Evidenced-based practices for enhancing recruitment and retention among Special Populations

The inclusion of special populations helps healthcare researchers succeed in improving health outcomes for everyone. Because people not appropriately treated for a variety of conditions do not benefit from the advances made elsewhere, significant healthcare costs are incurred, especially among children from low-income families [26,27]. For these reasons, the inclusion of special populations provides opportunities for health improvements that are not easy to predict but are certain to occur.

The NIH has attempted to address these inequities through its efforts to define special populations. As a first step, the NIH requires reporting metrics for women, children, and under-represented minorities who are participants in research studies (NOT-OD-16-010: Inclusion of Children in Clinical Research: Change in NIH Definition; NOT-OD-15-089: Racial and Ethnic Categories and Definitions for NIH Diversity Programs and for Other Reporting Purposes). This issue is further addressed by considerations of autonomy, beneficence and justice, as described by the Belmont Report, which stipulates that subjects should not be excluded from participation in a clinical trial simply because it is easier and more convenient to recruit participants from an urban, academic health science center [28]. These principles call upon investigators to consider undertaking clinical trials that allow the inclusion of special populations for reasons that are scientifically justifiable. The diversity of clinical trial needs, and the populations they might serve, cannot be easily met by individual investigators who may be forced to work outside of their scope of clinical or scientific expertise.

## 3. Approaches for recruiting special populations

## 3.1 Implementation and design

Research design has been identified as a key-influencing factor for making research more inclusive. Firstly, there are many types of research studies varying from FDA regulated clinical trials to community-based observational studies. Interest and participation is likely to vary based on the types of studies. A clinical trial that has an intervention arm with a placebo may be unattractive to potential participants because they believe that the intervention to be tested is better and they don't want to be randomized (UNM CTSC research participants, personal communication). There are trial designs, such as cross-over, stepped-wedge, and others that offer participants and communities increased access to study interventions. Including community consultation in early phases, and on specific design and implementation procedures has been shown to be an effective means of increasing trial and research study awareness, participation and enrollment.

Operationally, attention to both general and specific details in the implementation of a study can strongly affect recruitment and retention of individuals from special populations. Townsley et al., Selby & Siu [29] argue that many barriers to recruitment are created by researchers themselves. Logistical accommodations in research implementation have also been shown to significantly impact diversity inclusion in health research. Creating more frequent feedback loops for tracking recruitment rates can allow researchers to adjust approaches and modify materials to improve inclusion (Alexander et al., Table 2). Identifying a site champion to monitor and promote recruitment can enhance diversity of participation, as long as the target populations are appropriate to address the underlying scientific questions [30]. Ensuring that recruitment materials and research instruments are at an appropriate literacy level facilitates participation [17,31]. Establishing a personal connection between participants and research staff through follow-up calls, including caregivers or family members in the research, or even by sending birthday cards to participants in longitudinal studies, can strengthen retention [18,31,32] (Young, Table 2). Providing logistical support such as transportation [30-33], childcare, and creating flexibility in the time or location of research appointments [18,31] have potential to make it more likely that participants will be able to participate. And importantly, adequately munerating participants for their time and ensuring that compensation re is culturally appropriate are also important components of the process [17,18-31,34-39].

Trent et al. [40] suggest that special populations can be successfully recruited with "sufficient investment in the design and infrastructure of the study," and Townsley et al, Selby & Siu [29] found that provision of personnel and resources to accommodate the unique requirements of their target special population helped to remove barriers to recruitment. In general, a less rigid study design and logistical orientation toward the target group can promote recruitment and retention among special populations. Flexible study design can allow adaptation to the specifics of the target group [41].

## 3.2 Identification of trust issues among ethnic minorities

In retrospective analyses, members of African American, Hispanic, Asian, and Native American populations is frequently not mentioned in clinical trials reporting metrics, and if they are identified, representation from these groups are below expectations [42,43]. Depending upon the population being studied, members of under-represented minorities may not understand the concept of a clinical trial or have concerns that the research procedures may not be covered by insurance, which may include additional visits for medical care, travel costs, or laboratory tests. Furthermore, literacy rates or a primary language other than English can pose significant barriers to clinical trials participation [44]. This problem is even further accentuated for special populations, particularly those of low socioeconomic or minority status, and in both younger and older groups.

The challenges of recruiting minorities for clinical research have been well documented in the literature, and from our experiences working with special populations in the State of New Mexico [45-47]. Some studies show that rates of minority enrollment and participation in observational studies are similar to that by non-minorities [48-50], yet evidence suggests that there are significant barriers to participation for minorities in clinical trials [51]. Factors associated with non-participation and as a result of poor recruitment include: mistrust of researchers and government agencies [52,53], discomfort with the idea of being a "guinea-pig" [54], time and scheduling demands [55], economic barriers related to time off work [56], being excluded due to existing medical problems [57], and transportation to and from the research site [58]. Community barriers include fear of exploitation, being treated poorly, and low levels of knowledge regarding the need for medical research [54,59,60]. Many people and patients do not perceive any benefits (especially from non¬intervention studies) from research participation [61,62], and lack of incentives, especially financial incentives has been shown to reduce interest, recruitment and retention rates among low income and minority patients [38].

## 3.3 Accommodating culture and context

**Figure 2.**
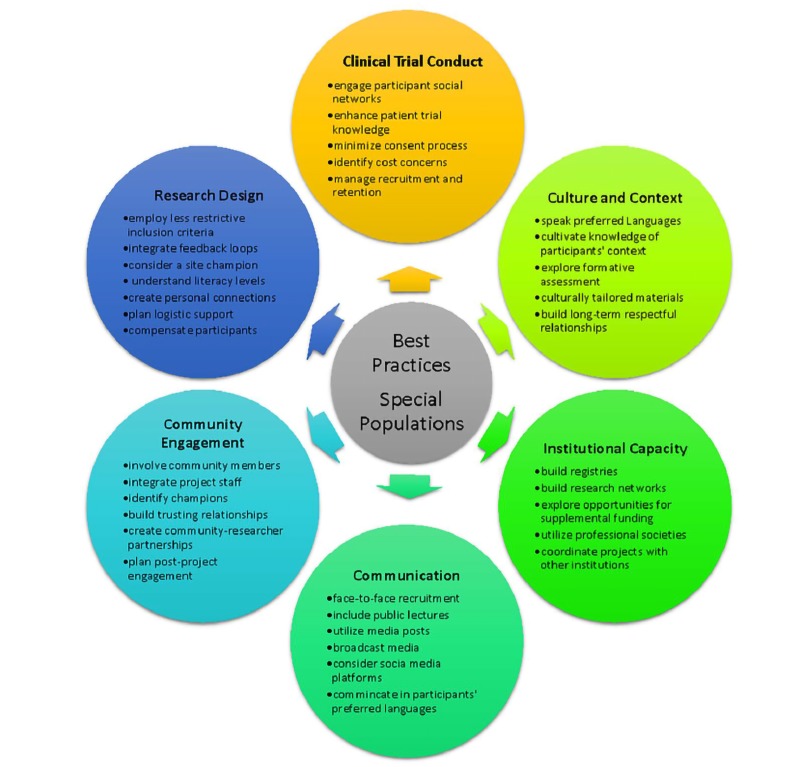
Summary points for improved clinical trials accrual and retention for participants from special populations.

Working with special populations in research can require that unique accommodations be made for the specific cultural and contextual realities of participants' lives (Figure 2, Table 2). It may be necessary to conduct a formative assessment to characterize the population of interest and to identify barriers to recruitment and participation [3,30]. Interviews with key community members or focus groups including members of the special population can be part of this process. Developing culturally tailored materials and protocols have been shown to improve recruitment and retention [3,33,63]. Language is a key dimension of culturally appropriate recruitment strategies for some special populations and should not be underestimated [18,31]. Recruitment materials available in the participant's language as well as ensuring members of the research staff are fluent in that language can go a long way to enhance recruitment and retention. A nested recruitment design can embed targeted recruitment strategies within a general recruitment plan in order to enhance participation by targeted special populations [3].

However, as Trickett [64] suggests, culturally targeted research strategies need to go beyond language or recruitment materials with images of individuals from the population of interest. Research design needs to be culturally and contextually "situated" to appropriately accommodate the participants' reality. Researchers and the scientific process will benefit from better understanding of participants' social, economic, and cultural contexts. This can include simple things such as knowing how and when to employ culturally appropriate forms of address [31], ways of asking questions [33], or knowledge of relevant holidays and religious observances or more complex culturally and contextually based perspectives, beliefs, behaviors, and experiences. Hiring project staff who are culturally matched to the population of study [19,30], such as Community Health Workers (CHWs), *Promotoras,* or community representatives who can operate as culturally competent insiders can significantly improve research team cultural competence and consequently improve participation by members of some special populations [18,31,65,66].

## 3.4 Outreach and communication

Communication is an important dimension of the recruitment and retention equation for special populations [32,33]. Outreach to the community through public lectures and strategically placed media spots can increase awareness of the significance of the topic of research [17,31,34], which can lead people to become interested in participating. Communications, such as brochures, posters, and informed consents, need to incorporate health literacy —not just grade level assessment—to enhance communication efficacy.

Through effective communication, researchers can also help participants understand how their participation in clinical trials research could be of benefit not only to themselves but to the broader community [31]. Participant altruism has been shown to be an important motivator for individuals from many special populations [18]. Communicating the expanded informational context means that when a study is implemented and in the recruitment phase potential participants will have an increased likelihood of being interested in participating.

## 3.5 Participant awareness

Community-engaged research practices [67] and Community-Based Participatory Research (CBPR) approaches [68] have the capacity to reveal complex community dynamics that need to be considered in health research. As such, community engagement and participation have been shown to improve the scientific quality of the research, the cultural competence of researchers, and recruitment and retention of participants [69]. Involving community members in designing and implementing a research study through reviewing instruments, working as project staff [31,33], or identifying individuals from the population of study who can help build relationships and conduct outreach in the community [18,30], can encourage recruitment. Community-engaged research processes and the attendant relationships that are established can also help to overcome entrenched mistrust of researchers or the research process that exists among some special populations. Ford *et al.* [44] and George et al. [18] found that participants preferred research conducted in community contexts, similar to the findings of others [17,18,70].

The "relationship" and its components, like trust, cooperation, power, and risk perception, between researchers and the community is particularly key [19,22]. A commonly reported perspective in the community is that researchers only show up when they want to get people to participate in their study. Killien et al. [19] recommends that "productive partnerships between researchers and community members should be encouraged to continue beyond the life of the specific research project" for researchers to overcome being seen as "taking" from the community. To overcome community perceptions of mistrust directed against the government, Ejiogu et al. [53] developed door-to-door outreach efforts, neighborhood meetings, and mobile exam centers to increase enrollment for African Americans who participated in a longitudinal aging study. They also provided certificates of confidentiality and safety training programs that involved the local police. Newman [71] overcame many of these same barriers by addressing the risks of clinical trial participation and the trial's risks from a family perspective. Additionally, using partner-led recruitment allows community organizations collaborating with researchers to use techniques that researchers may not be familiar with and to leverage existing relationships of trust to identify, outreach, and motivate individuals to participate [19,22,72].

Recruitment of special populations in rural communities is especially hard, given geographical location challenges, transportation barriers, and lower numbers of eligible participants [73]. Lack of awareness of research opportunities is an additional barrier to successful involvement of rural communities in clinical research. Community-based recruitment strategies could increase participation among rural community participants. Utilizing community liaisons to help in the recruitment of rural participants can prove an effective strategy for improving researcher-participant trust, research awareness, and address geographic challenges associated with access to care. Furthermore, many potential research subjects living in rural locations may have never heard of clinical trial opportunities, further distancing them as participants. Successful efforts to increase participation have centered on community awareness, mobile recruitment sites, and involving research participants in interventions.

## 3.6 Families, parents and children

For clinical trials that are intended to recruit children and adolescents, federal regulations mandate that the consenting process include language that is appropriate for research subjects that fall into the 7 to 11-year-old and 11 up to age 18-year old assent categories and be written in languages that are understood by the children and their parents. Clinical trials that have successfully recruited and retained minors as participants utilized social media networking techniques, provided increased incentives, including money and gifts, and focused on flexibility to accommodate the needs of working parents [74,75]. Wiemann et al. [76] considered the participants' mothers important points of contact, while Zamora et al. [77] used culture-specific and parent-centered approaches to be important aspects of protocol recruitment success. In a large study of children with genetic risk of type-1 diabetes, researchers assessed factors associated with poor retention among ethnic minorities [74]. They found that it was essential to solicit multiple types of contact information since many families were often mobile. In a follow-up study, researchers developed a "high risk of early drop out" score that targeted the group with a higher score for retention interventions [78]. They evaluated differences in the groups by intervention (vs. none) and by risk score (high vs. low). Withdrawal rates were significantly higher in the high-risk compared with the low-risk groups who did not get the intervention, while withdrawal rates were lower in the high scoring group that participated in the intervention compared to those who did not. In the intervention group, there was no significant difference between the high and low risk groups for early withdrawal. This study did not evaluate a systematic intervention but allowed interventions to be designed by individual sites. The most common intervention approaches to reduce attrition included: increasing individual attention for consistency of interaction, enhancing family engagement, and hiring a retention coordinator to increase intensity and consistency of patient contacts. Many studies implement recruitment and retention strategies similarly with an eye toward addressing the barriers and facilitators "organically" (Table 2). Cui et al. [75] reviewed strategies used in studies of minority and low-income children in trials that included obesity-related behavior modification (not outcomes). They found that of 43 studies, 25 (55%) reported which strategies were used. The most common were: increasing incentives, including money and gifts, drawing for gift cards, rewards for retention, including cash, food, exercise equipment, recipe books, YMCA memberships holding family nights and having strong community connections. In each case, studies emphasized the need for appointment reminders and follow-up calls.

Community connections are emphasized in many studies but few define it well. Some defined approaches include having a community advisory board, interacting with neighborhood groups, churches, and schools. Study flexibility, resources that address access barriers such as language, transportation, child-care, and time away from work were needed.

## 3.7 Fair compensation

Evidence for strategies to enhance recruitment and retention of special populations is varied. The best evidence includes assessments in trials and experimental designs, as well as systematic reviews [39,79-81]. In a systematic review, Brueton et al. [82], examined eight trials that randomized evaluations to improve response and retention rates (most embedded in trials). Factors shown to be associated with higher response rates Distributed under creative commons license 4.0 included monetary incentives, higher value (more money) incentives, as well as shorter surveys (Table 2). They note a lack of evidence for: type of postal delivery, non-monetary incentives, donations to charity, and sending surveys out early. In a separate systematic review, Treweek et al. [80] assessed methods to improve recruitment into randomized controlled trials (Table 2). Results from 45 trials showed that most effective interventions included: telephone reminders, using opt-out procedures for contacting potential participants, open trial designs, and payment for participation.

Payment for research emerges as one of the most important strategies with respect to engaging and retaining special populations in research. Payments that are non-conditional were found to be more effective than 'conditional' ones. In a study of 21 to 65-year old participants, Alexander et al. [35], evaluated clinical trials enrollment that was influenced by incentive combinations, including no incentive, conditional (promised), and unconditional incentive. They found that the highest enrollment was among those who received unconditional cash incentives, and that retention was linked to higher value incentives. While this study did not recruit members of special populations, per se, the findings reflect important considerations for all study participants. Financial incentives have shown to be more effective in adolescents attending STI clinics [37], ethnic minority men-who-have-sex-with men (MSM) recruited for HIV vaccine trials [71], and reducing loss to follow-up in women who inject drugs during interventions [83]. While most researchers found that compensating participants for their time was extremely important in trial recruitment and retention, in a survey of 2,150 nationally representative adults Walter et al.[38] found that requested payments differed significantly by racial/ethnic group, with Hispanics requesting more payment than non-Hispanic Whites (Table 2) [38].

## 3.8 Improving the consent process

Proper informed consent is a process of information exchange between researchers and participants to gain voluntary agreement to consent [84,85]. Efforts to increase participation in clinical studies must include a dynamic consent conversation, which must take into consideration whether the potential research participant understands the scientific objectives of the study. Because "clinical trial" is a term that is often unfamiliar in under-represented communities, efforts to educate potential participants were met with success in several studies [86]. Education about the process of participating in a clinical trial, especially regarding the consent process, improved enrollment. Efforts to educate participants in a culturally-sensitive manner, including why the subject's ethnic group were employed to increase participation in a study to better understand the attitudes of a healthy population towards genetic determinants of health. Matsui et al. [87] found that their efforts to better inform participants about the risks and benefits of a genetics study using DOI: http://dx.doi.org/10.18053/jctres.04.201801.003 a re-iterative consenting and follow-up process resulted in a lower participation rate than in the control population. However, there were fewer withdrawals from the experimental study population than for the control group, suggesting that those who participated in the study were better informed and more committed to completing the study activities [87]. The research group also found that study participants who had more time to consider being in the study were more likely to complete the study activities [88]. Their efforts to educate study participants using an on-going consent process—to instill a genuine partnership based on cooperation—show that informed consent is a time and education-dependent process. Researchers involved in special population studies are especially impacted by these findings, because they must often overcome mistrust, misperceptions, and misunderstandings from past special populations research.

## 3.9 Structural and institutional considerations

While researchers can take study-specific action to improve representation of special populations in health research, there are also institutional and structural approaches that deserve more attention from the research community. Napoles & Chadiha [3] suggest creating registries for individuals from special populations who express interest in participating in research. Such registries could be site-specific, or they could be cross-site with infrastructure costs of maintaining the registry shared by different sites or teams. Research networks can also be influential in creating cross-site relationships for recruitment [32]. Establishing ongoing collaboration with organizations and agencies that interface with members of a special population can help researchers identify and connect with participants, and individuals who work for these organizations are often important allies in decreasing participant mistrust [31].

Yet beyond institutional capacity-building, there are also structural factors outside the control of research teams that influence recruitment of special populations. Ford *et al.* [17] suggest that the cultural diversity of the research team must be considered, but the continuing lack of diversity means that it is often difficult to create a legitimately diverse research team with the capacity to reach special populations. Napoles & Chadiha [3] write about the lack of funding available for conducting research on recruitment diversity challenges and the fact that Funding Opportunity Announcements (FOAs) designed to specifically address this issue are few and far between. They suggest that in lieu of specific FOAs, funders could provide opportunities for supplemental funding to improve recruitment for special populations within the context of broader studies. And, significantly, what is clear from the complexity of the issues involved in recruiting and retaining individuals from special populations to participate in research, researchers need to allocate more time for planning the design and implementation of studies that include special populations [31,32]. Funders should also be made aware that working with special populations often requires extended time frames and will require the allocation of resources at a level appropriate for such research and its dissemination [89].

Practitioners face a different set of barriers regarding participation in clinical trials. These barriers can be system-related, including lack of time and inadequate research experience. In addition, practitioners participating in clinical trials hardly ever receive recognition for their efforts or receive adequate incentives. To encourage participation, adequate incentives should also be considered for clinic staff, and these can include non-financial incentives such as continuing medical education credits.

## 4. Summary points: ask the right questions, do the right things

Despite the 1993 National Institutes of Health (NIH) Revitalization Act [90] requirfiging that NIH-sponsored clinical research include women and members of minorities and their subpopulations, special populations are not being appropriately invited or recruited for research. Effective engagement is an important strategy for the successful recruitment of participants. Comparing study designs using passive versus active recruitment methods are effective measuring strategies for what works in the recruitment of underrepresented minorities. Active recruitment involves targeting specific special populations and targeting participants in person, by phone, or by mail. Passive recruitment informs the community about a research project through flyers and brochures, prompting research participants to contact research staff [91]. Evidence shows that less than 10% of patients participate in trials [91], and according to the Department of Health and Human Services, only 12% of U.S adults possess proficient health literacy [92]. It is important to emphasize the need to employ multidimensional strategies to improve inclusivity. The approaches described in this monograph can help researchers improve targeted special population recruitment in clinical trials. Building trust, conducting trials that matter, and offering attractive incentives as well as offering easy opt out options, are some of the best practices identified for this purpose (Table 2; Figure 2). What are the best practices for better clinical trials?

## 4.1 Development of clinical trials that matter to the special population participants

A key influencing factor for special population recruitment is developing clinical trials that match the health priorities of communities of study or target populations. While much enthusiasm for a clinical trial might exist among the investigators, it is very unlikely that the intended participants will consent to enrollment if the trial is of little or no interest to the subjects themselves.

## 4.2 Utilization of meaningful incentives

Research requires work, especially for the research participants. Inadequate incentives and/or poor logistical planning is enough to discourage participation. As motivators, incentives have to be commensurate with the time and effort potential participants have to take to prepare and commit in trial participation and easily facilitate access to the study site. Non-conditional incentive payment for research is one of the most important strategies for engaging and retaining special populations.

## 4.3 Building trustworthy relationships takes time and effort

Respecting the privacy and wishes of the community is an important factor when recruiting special populations. The conduct of clinical trials matters greatly to many stakeholders, including the principal investigators, the institutions that help to sponsor the research, and the regulatory agencies that oversee their conduct. But the most important stakeholders are the research participants themselves. Without their trust, little progress will be made, but with their trust, great progress will continue to be towards improved health outcomes for all.

The current economic landscape of healthcare continues to challenge community hospitals and academic health systems alike [93]. As large healthcare systems undergo mergers or acquisitions, research practices are impacted in ways that are not fully understood. Newly formed partnerships between private and public institutions bring together different cultures in business practices, missions, and infrastructures related to referral patterns, research capacities and overall healthcare objectives. Nevertheless, demographic changes across the US will compel the healthcare industries of the 21^st^ century to embrace the healthcare needs of our special populations, which
